# Prevalence and Initial Diagnosis of Cerebral Palsy in Preterm and Term-Born Children in Taiwan: A Nationwide, Population-Based Cohort Study

**DOI:** 10.3390/ijerph18178984

**Published:** 2021-08-26

**Authors:** Hsin-Hua Wang, Yea-Shwu Hwang, Chung-Han Ho, Ming-Chi Lai, Yu-Chin Chen, Wen-Hui Tsai

**Affiliations:** 1 Division of Neonatology, Department of Pediatrics, Chi Mei Medical Center, Tainan 71004, Taiwan; b101092111@tmu.edu.tw (H.-H.W.); ruby721222@hotmail.com (Y.-C.C.); 2 Department of Pediatrics, Chi Mei Medical Center, Liouying, Tainan 73657, Taiwan; 3 Department of Occupational Therapy, College of Medicine, National Cheng Kung University, Tainan 70101, Taiwan; yshwang@mail.ncku.edu.tw; 4 Department of Medical Research, Chi Mei Medical Center, Tainan 71004, Taiwan; ho.c.hank@gmail.com; 5 Department of Information Management, Southern Taiwan University of Science and Technology, Tainan 71005, Taiwan; 6 Division of Pediatric Neurology, Department of Pediatrics, Chi Mei Medical Center, Tainan 71004, Taiwan; vickylai621@gmail.com; 7 Department of Pediatrics, Chi Mei Medical Center, ChiaLi, Tainan 72263, Taiwan; 8 Graduate Institute of Medical Sciences, College of Health Sciences, Chang Jung Christian University, Tainan 71101, Taiwan

**Keywords:** preterm, cerebral palsy, prevalence, diagnosis, low birth weight

## Abstract

The aim of this long-term longitudinal study in Taiwan was to estimate and compare the prevalence of cerebral palsy (CP) and to identify the age of CP diagnosis of term-born and preterm children with different birthweights. Records of 1494 extremely low birth weight (ELBW, <1000 g), 3961 very low birth weight (VLBW, 1000–1499 g), 19,612 low birth weight (LBW, 1500–2499 g) preterm, and 100,268 matched term-born children were retrieved from Taiwan′s National Health Insurance Research Database. According to a 12-year retrospective data review, the results showed the highest prevalence of CP in preterm ELBW children (147.3 cases per 1000 neonatal survivors), followed by preterm VLBW (97.2 cases), preterm LBW (27.7 cases), with the lowest prevalence in term-born children (2.5 cases). Regardless of the birthweight group, 90% of preterm children with CP were diagnosed by 4 years of age, but it was 7 years before 90% of term-born children with CP were diagnosed. After removing the children whose CP was caused by brain infections, injuries, or cerebrovascular accidents after 4 months of age, there were similar mean ages at the initial CP diagnosis (1.58–1.64 years of age) across birthweight groups born prematurely, but initial diagnosis occurred at an older age (2.41 years of age) in term-born children. The results indicate that birthweight is reversely correlated with the prevalence of CP in preterm children. Although the three preterm birthweight groups received different types of developmental follow-up programs after birth, it did not influence their age at the initial diagnosis of CP. Furthermore, we suggest that follow-up for at least 4 years after birth for preterm children, and 7 years for term-born children, is optimal for estimating CP prevalence. In order to identify and provide early intervention for term-born children with CP earlier, it is suggested that parents routinely fill out a self-reported motor developmental screening questionnaire and pediatricians conduct a motor developmental examination on term-born children at each time of scheduled vaccination injections.

## 1. Introduction

Cerebral palsy (CP) describes a group of disorders characterized by movement and postural problems, which are attributed to non-progressive injuries in the developing brain [1]. Among neonatal survivors with CP, approximately 30–40% of them are born prematurely [2]. The most common cause of the preterm population being at a high risk of CP is a white matter injury (e.g., periventricular hemorrhage and periventricular leukomalacia) during the perinatal period [3,4]. The main causes of CP for those born at term are thrombophilic disorders (e.g., cerebral infarction related to perinatal or intrauterine thromboembolism) [4,5], kernicterus, perinatal hypoxic-ischemic event, TORCH infection, brain malformations, and perinatal ischemic strokes [6,7].

Although a large number of studies have investigated the prevalence of cerebral palsy in preterm populations, most of them are based on the data of Western countries, including various European countries, Canada, Australia, and the United States of America [8]. In contrast, similar studies on preterm children in Asian countries are relatively fewer and with smaller sample sizes [9], except for one Korean population-based nationwide study, which investigated the rate of preterm birth in children with CP [10].

The results available on preterm populations consistently indicate that the prevalence of CP increases with decreased gestational age or birthweight. However, even for preterm infants at a similar gestational age or birthweight range [8,11], the estimations of their prevalence of CP significantly varies across studies [8,11]. Different ages of follow-up used in the previous studies (i.e., ranging from 8 months to 10 years of age) may be one of the reasons for the lack of agreement on the prevalence of CP in preterm children [8,12,13]. For example, in a study following up at two years of age, researchers reported the prevalence of CP for extremely low birth weight (ELBW) infants was 69.7 per 1000 survivors [14]. However, the prevalence of CP was estimated by Salokorpi et al. [12] to be 190 per 1000 for the same birth weight survivors when they follow up to four years of age. There is limited evidence available for determining the optimal number of follow-up years required to accurately estimate the prevalence of CP in preterm and term-born populations. The minimum follow-up age used by the Surveillance of Cerebral Palsy in Europe (SCPE) for a confirmed diagnosis of CP is 4 years of age [15], and 5 years of age is used by the Australian Cerebral Palsy Register (ACPR) [16]. It is possible that studies with shorter lengths of follow-up may tend to underestimate the prevalence of CP in preterm and term-born populations. Therefore, a long-term, longitudinal study on the emergence of CP diagnosis for preterm and term-born children is needed to provide an evidence-based guide for this issue.

Early diagnosis of CP is certainly important for increasing the success of intervention and decreasing the impacts of the disorder. To date, little is known about whether the age of initial diagnosis of CP may be caused by different follow-up programs provided for children at different levels of maturity at birth and/or the alertness of health professionals to the condition. In Taiwan, since 1995, preterm infants born with a birth weight below 1500 g have received a comprehensive, interdisciplinary (i.e., pediatric neurologists, neonatologists, rehabilitation experts, etc.) follow-up program at 6, 12, and 24 months of age, which, in 2006, was extended to 5 years of age [17]. However, preterm infants who weigh more than 1500 g at birth generally receive a routine brief developmental examination in hospitals by neonatologists after discharge at least until they reach 2 years of age. Children born at term go to a hospital or clinic for scheduled vaccination injections after birth, but they may or may not receive a developmental check-up by pediatricians at that time. Additionally, term-born children with CP, particularly those with mild motor impairments and without risk factors (e.g., complicated birth or brain insult findings) may be difficult to diagnose at an early age [2]. Some clinicians adhering to a “wait and see” approach may delay the diagnosis of CP in these children [2]. Therefore, the influence of different developmental follow-up programs for preterm and term-born children and levels of prematurity at birth on the age of initial diagnosis of CP requires investigation.

In this study, we used a long-term (12 years) national population dataset to estimate the prevalence of CP in Taiwanese children born prematurely and at term. There were three purposes in this study. First, we aimed to identify the prevalence of CP in term-born and preterm children with different birthweight ranges (LBW, VLBW, and ELBW) in Taiwan. Secondly, we aimed to investigate the relationship between the prevalence of CP and ages at follow-up in preterm and term-born children which may answer the question as to how many follow-up years after birth are adequate to accurately estimate the prevalence of CP in both populations. Finally, we aimed to realize the influence of different follow-up programs on the age at initial diagnosis of CP. Therefore, we compared the age of initial CP diagnosis in term-born and preterm children after excluding those children with acquired CP which was caused by brain insults after 4 months old. Four months of chronological age was chosen because many premature infants, particularly for ELBW infants with CP, may not be discharged from hospital until 4 months of chronological age. If a cut-off age earlier than 4 months for exclusion was chosen, many preterm ELBW infants with CP, caused by intraventricular hemorrhage, periventricular leukomalacia, or meningitis during hospitalization, might also be excluded.

## 2. Methods

### 2.1. Source of Data

The database used for the present study was retrospectively retrieved from the medical claim files of Taiwan′s National Health Insurance Research Database (NHIRD) provided by the Bureau of National Health Insurance (BNHI). The NHIRD provides all inpatient and ambulatory medical claims for approximately 99% of Taiwan′s residents [18]. To ensure the accuracy of claim files, the BNHI performs quarterly expert reviews on a random sample of every 50 to 100 ambulatory and inpatient claims [19]. Therefore, the information obtained from the NHIRD is considered complete and accurate [20,21]. In this study, all data were coded using the International Classification of Diseases 9th Version Clinical Modification (ICD-9-CM). We retrieved ambulatory care claims, inpatient claims, registry for beneficiaries, registry for medical specialties, and registry for patients with catastrophic illness from the NHIRD datasets. Access to research data was reviewed and approved by the Review Committee of the National Health Research Institute, Taipei, Taiwan (NHIRD-105-052) and the Institutional Review Board of Chi Mei Medical Center, Tainan, Taiwan (10410-E09).

### 2.2. Study Design and Identification of Study Subjects

**Figure 1:**
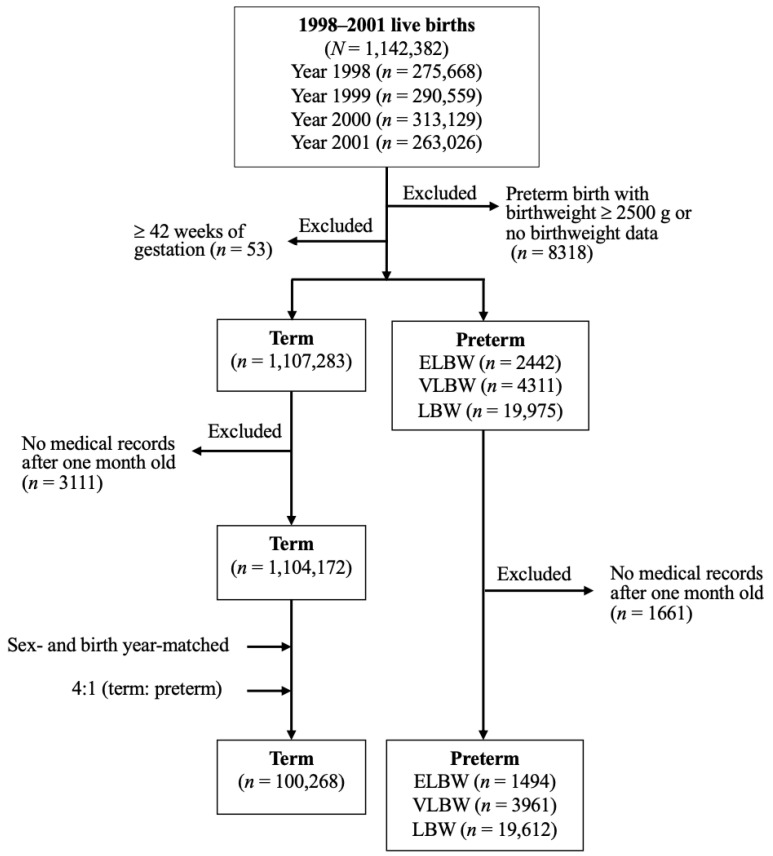
Cohort flowchart. ELBW: extremely low birth weight; VLBW: very low birth weight; LBW: low birth weight.

This was a retrospective population-based cohort study based on a cohort of all live births occurring from 1998 to 2001. Live preterm births with discharge codes of extremely low birth weight (ELBW) (<1000 g, ICD-9-CM 765.01, 765.02, or 765.03), very low birth weight (VLBW) (1000–1499 g, ICD-9-CM 765.14 or 765.15), and low birth weight (LBW) (1500–2499 g, ICD-9-CM 765.16, 765.17, or 765.18) were identified as the preterm groups. Preterm children without any medical records after one month old (i.e., died or moved out of Taiwan) were excluded (Figure 1).

The term-born population used as the control group was defined as those without the codes of preterm birth (ICD-9-CM 765) and post term (gestational age ≥ 42 weeks, ICD-9-CM 766.2). Similar to the protocol for preterm children, term-born children without any medical records after one month old were also excluded (Figure 1).

### 2.3. Definition and Diagnosis of Cerebral Palsy

We sequentially reviewed the data for the present cohorts from birth to 12 years of age, searching for the children with the code of infantile cerebral palsy (ICD-9-CM 343). To further ensure the accuracy of the diagnosis, the children must have been diagnosed with infantile CP by pediatricians or rehabilitation physicians for at least three outpatient department visit claims within a year. Additionally, because CP by definition excludes children with progressive conditions, those children meeting the above criterion but with the ICD-9-CM codes of progressive neurological disorders (330, 331.0–331.2, 331.7–331.9, 334, 335, 340, 341) or spina bifida (741) later were excluded from the CP group.

### 2.4. Statistical Analysis

In the statistical analyses, a comparison was made of the sociodemographic characteristics of the preterm and term-born groups using a Pearson′s chi-square test. Then, a life-table method [22] was used to estimate the specific cumulative incidence rate of CP during the follow-up period. Furthermore, the Cox proportional hazards regression model was used to determine the independent effect of birth weight on the risk of CP, with the term-born group as a reference group after adjusting for age, sex, geographic area, urbanization status, and insured salary grade. Subjects who died in the hospital or those with clinical outcomes that were not of interest were censored in the survival analysis. The censoring date was the date of death, or if the participants did not die in the hospital during the follow-up, the censoring date was either the date of their last withdrawal from NHI or the date of termination of the study, i.e., 31 December 2013. The distribution by age at first diagnosis of cerebral palsy in terms of preterm and term-born births was illustrated using a box plot with a Kruskal–Wallis test used to compare the differences. All statistical analyses were performed with SAS (version 9.4, SAS Institute, Cary, NC, USA). A *p*-value of <0.05 was considered to be statistically significant.

## 3. Results

According to Taiwan’s National Health Insurance Research Database, the total number of births from 1998 to 2001 was 1,142,382. After excluding children without medical records after one month of age, we identified 1494 ELBW, 3961 VLBW, and 19,612 LBW children born prematurely. A total of 1,104,172 term-born neonatal survivors matching the criteria were identified. Then, through a sex and birth-year matching process for preterm children with a ratio of 4:1 (term/preterm), 100,268 term-born children were randomly selected as the control group.

The demographic characteristics of the children are presented in Table 1. There was a significant group difference in the proportion of children in the demographic variables (sex, residence areas, and insured salary grades). A significantly higher proportion of girls and a greater proportion of children lived in South and East Taiwan in the preterm VLBW and ELBW groups compared with the term-born group. In addition, a significantly higher proportion of families were in the lowest insured salary grade in the three preterm birthweight groups than the term-born group.

Table 2 describes the prevalence and adjusted hazards ratio (AHR) of CP for the term and preterm children. The prevalence of CP was 2.53 cases per 1000 neonatal survivors for the term group, 27.69 cases for preterm LBW, 97.20 cases for preterm VLBW, and 147.26 cases for preterm ELBW. When using the term group as a reference, the adjusted hazards ratio (AHR) of CP was 11.08 (95% CI 9.54–12.86) for the preterm LBW group, 40.40 (95% CI 34.48–47.34) for the preterm VLBW group, and 62.73 (95% CI 52.37–75.14) for the preterm ELBW group.

**Figure 2:**
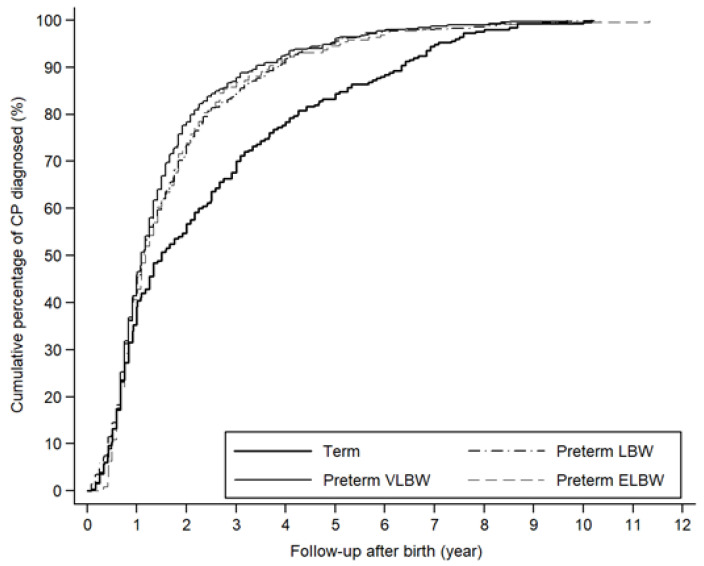
Cumulative percentage of children with cerebral palsy with different follow-up years after birth in preterm and term-born cohorts. CP: cerebral palsy; ELBW: extremely low birth weight; VLBW: very low birth weight; LBW: low birth weight.

There was a similar trend in the relation between follow-up years and accumulated percentage of CP diagnosis for the birthweight groups born prematurely. Approximately 90% of preterm children with CP were diagnosed by 4 years of follow-up after birth. However, in the term-born children, it required 7 years of follow-up to reach 90% of the prevalence of CP (Figure 2).

**Figure 3:**
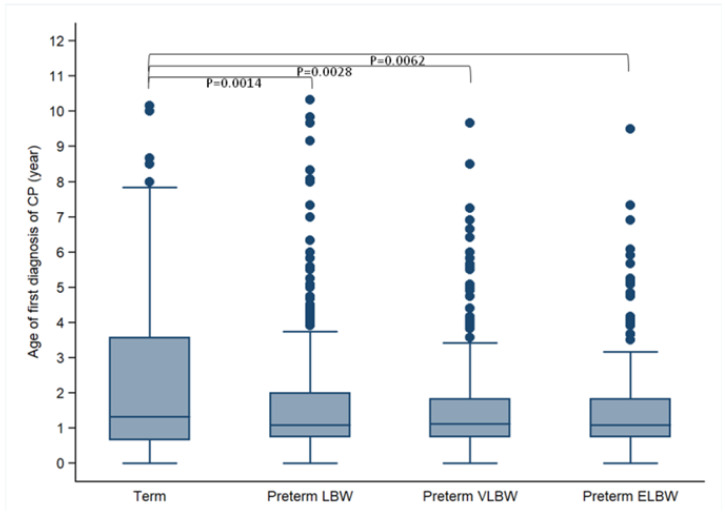
Comparison of age at first diagnosis of CP in preterm and term-born children whose CP was caused by brain insults before 4 months of age. The median age at first diagnosis of above groups presented as 1.33 (interquartile range, IQR: 0.67–3.58) for the term group, 1.08 (IQR: 0.75–2.00) for the preterm LBW group, 1.12 (IQR: 0.75–1.84) for the preterm VLBW group, and 1.08 (IQR: 0.75–1.83) for the preterm ELBW group. CP: cerebral palsy; ELBW: extremely low birth weight; VLBW: very low birth weight; LBW: low birth weight.

After removing the children whose CP was caused by brain infections, injuries, or cerebrovascular accidents after 4 months of age, the age at first diagnosis of CP in the term and preterm children is presented in Table 3 and Figure 3. The mean age at first diagnosis of CP for the term group was significantly greater than that for all preterm groups (2.41 vs. 1.58–1.64 years of age, *p* = 0.0064); however, it appeared to be consistent among the three preterm groups (Table 3 and Figure 3). Overall, among the preterm children with CP, 75% of them were diagnosed by approximately 2 years of age, but first diagnosis of CP was delayed to approximately 3.5 years of age for term-born children. Similarly, 90% of preterm children received diagnosis of CP by approximately 4 years of age, but for the term-born children, 90% received diagnosis of CP by more than 6 years of age (Table 3).

## 4. Discussion

In this national- and population-based study, we retrospectively investigated the prevalence of CP and the age of first diagnosis of CP for term and preterm children from birth to 12 years old. Consistent with the findings reported in previous studies [8,16], for Taiwanese children, the prevalence of CP was also significantly higher in the preterm children than in those born at term. Additionally, similar to previous studies [8,11], we also found that there was a reverse relationship between birth weight and the prevalence of CP in the preterm children. However, the prevalence of CP in preterm and term groups in the present study appeared to be higher than the pooled prevalence reported in previous review studies [8,11]. The differences may be explained by the methodology used in our study and others’, such as age at follow-up, sample size, birth years of cohorts, selection criteria of participants (e.g., neonatal survivors vs. live births), and the source of the database (e.g., administrative vs. patient registry database). Studies have shown that larger sample sizes [11], earlier years of birth [16], using the number of neonatal survivors instead of live births as the denominator of the estimation [16], and using administrative instead of patient registry database [23] may lead to a higher estimated prevalence of CP. Our pooled prevalence of CP (3.49 cases per 1000) of preterm and term groups was comparable with that reported by a study on the Taiwanese general population at 4 years old or older (3.2 case per 1000, 95% CI 2.8–3.7), which also used the same database as ours (i.e., Taiwan′s National Health Insurance Research Database) [24].

Our findings revealed that approximately 20% of preterm children with CP were not diagnosed by 2 years of age. Even after following up until 4 years of age, approximately 10% of preterm children with CP were not counted in the estimation (Figure 2). Therefore, studies using a shorter period of follow-up years at or below 2 years of age may have been at risk of underestimating the prevalence of CP in preterm children. In addition, our results indicated that longer follow-up years (i.e., about 7 years) may be required to obtain a more correct estimation of CP for term-born children.

The age at first diagnosis of CP in our preterm and term-born children was comparable with that reported in previous studies [25,26]. In our study, the median age (interquartile range) at first diagnosis was approximately 1.1 (0.8–2.0) years old in preterm children and 1.3 (0.7–3.6) in term-born children, compared with 1.5 (1.0–2.3) years old in very preterm children, 1.4 (0.9–2.4) in moderately preterm children, 1.6 (0.8–3.0) in late preterm children, and 1.5 (0.8–3.3) in term-born children in Finland’s national register study [26]. In the present study, by comparing the age at first diagnosis of CP in preterm and term-born children, we found that quite a few term-born children were first diagnosed with CP at a later age than those born prematurely. Approximately 10% of preterm children with CP were first diagnosed after 3.5 years of age, whereas up to 25% of term-born children with CP were first diagnosed after 3.5 years of age. There are two plausible explanations for this delay found in term-born children. Firstly, unlike preterm children, term-born children may not have a routine developmental check-up by pediatricians at the time of scheduled vaccination injections. In addition, parents would not expect a CP diagnosis for their child if they had taken home a term-born infant without any disease diagnosis from the hospital. It may not be until the child starts missing motor milestones that a motor disorder might begin to be suspected. Secondly, sometimes, early diagnosis of CP may not be easy, particularly for those children with mild motor deficits. Instead, pediatricians and rehabilitation physicians may tend to diagnose these term-born children as developmentally delayed in the early years. To improve early diagnosis and intervention for term-born children with CP, pediatricians should check a child’s motor development at each appointment for scheduled vaccination injections. In addition, we encourage parents of all term-born infants to fill out the developmental screening item questionnaire in the Children′s Health Booklet developed by the Health Promotion Administration, Ministry of Health and Welfare in Taiwan [27] when they bring their infants to receive routine vaccine injections. When children appear to have a delay in their motor development, they should receive a detailed assessment by pediatricians or rehabilitation physicians.

There were no significant differences in the initial age at diagnosis of CP across all birth weight groups in the preterm population. These findings imply that a history of preterm birth may suggest that pediatricians or rehabilitation physicians should pay additional attention to the neuromuscular signs and motor development of these children. Therefore, although only preterm children who are more mature and heavier at birth receive a brief examination in clinics, those with CP would be identified early. On the other hand, although the age at initial diagnosis of CP in our preterm and term children was similar to that reported in previous studies [25,26], it may be possible to bring the diagnosis of CP even earlier (e.g., before 6 months’ corrected age) with the routine use of MRI and suitable motor assessment tools for at-risk infants [28]. In addition, it is suggested that future studies investigate the initial age at diagnosis of other developmental disorders (e.g., autistic disorder spectrum and attentional deficit hyperactive disorder) across different gestational ages or birthweights in the preterm population.

A few limitations exist in the present study. There may be a lack of universal definitions of CP diagnosis among different physicians. Therefore, to increase the reliability of diagnosis, we set rigorous criteria for the retrieval of CP cases from the administrative database, including that the children required at least three outpatient department visit claims of a diagnosis of CP within one year, and only diagnosis by pediatricians and rehabilitation physicians were counted into the CP cases. However, the consistency and correctness of the criteria for diagnosis of CP in these pediatricians and rehabilitation physicians cannot be verified in the present study due to the nature of the NHIRD (i.e., the name of the physicians who made the CP diagnosis for the children of interest were de-coded). Interpersonal biases regarding the criteria for the diagnosis of CP may exist. In addition, the code of ICD-9-CM 765.2× for weeks of gestation was unavailable in our NHIRD for the cohorts born in the years of 1998–2001; therefore, we were unable to classify the present preterm cohort according to their gestational age at birth. Although the data regarding the subtypes of CP are available in our database, considering that the complicated criteria used to decide the subtypes of CP may influence the consistency of a physicians’ diagnoses, we did not further investigate the subtypes of CP in the present study.

## 5. Conclusions

The results of this study indicate that the prevalence of CP in Taiwanese children born prematurely and at term were higher than that reported in developed Western countries, which may be attributed to methodological differences (e.g., years of follow-up, selection criteria of participants, source of database). The results confirm that the prevalence of CP is related to birth weight in the preterm population, with the highest prevalence in the ELBW group, followed by the VLBW and LBW groups. The prevalence of CP in each preterm group was higher than in term-born children from 11 (LBW) to 63 times (ELBW). In addition, based on the present study, we suggest that the follow-up years after birth should be at least 4 years for preterm children and 7 years for term-born children to provide an optimal estimation of the prevalence of CP in these two populations. On average, term-born children were first diagnosed with CP at a later age than those born prematurely. To identify term-born children with CP at the earliest possible time, we encourage the parents of term-born children to routinely fill out a self-reported motor developmental screening questionnaire, and also suggest pediatricians conduct a motor developmental examination on term-born children at the time of each scheduled vaccination injection.

## Figures and Tables

**Table 1:** Characteristics of the preterm and term-born cohorts in Taiwan.

	*n* (%)				*p*
	Term	Preterm LBW (1500–2499 g)	Preterm VLBW (1000–1499 g)	Preterm ELBW (<1000 g)	
Total number (*n*)	100,268	19,612	3961	1494	
Sex			*	**	0.0005
Male	53,660 (53.52)	10,629 (54.20)	2040 (51.50)	746 (49.93)	
Female	46,608 (46.48)	8983 (45.80)	1921 (48.50)	748 (50.07)	
Region			**	*	0.0006
North	49,857 (49.72)	9779 (49.86)	1976 (48.89)	753 (50.40)	
Central	28,301 (28.23)	5633 (28.72)	1049 (26.48)	373 (24.97)	
South	16,865 (16.82)	3168 (16.15)	691 (17.45)	274 (18.34)	
East	5245 (5.23)	1032 (5.26)	245 (6.19)	94 (6.29)	
Urbanization					0.0655
Urban	52,615 (52.47)	10,505 (53.56)	2044 (51.60)	763 (51.07)	
Suburban	36,710 (36.61)	7022 (35.80)	1493 (37.69)	559 (37.42)	
Rural	10,943 (10.91)	2085 (10.63)	424 (10.70)	172 (11.51)	
Insured Salary Grade (NTD)		***	***	***	<0.0001
None	9706 (9.68)	3542 (18.06)	773 (19.52)	256 (17.14)	
Low (1–16,500)	23,562 (23.50)	4610 (23.51)	942 (23.78)	364 (24.36)	
Middle (16,501–33,300)	46,008 (45.89)	7879 (40.17)	1587 (40.07)	594 (39.76)	
High (>33,300)	20,992 (20.94)	3581 (18.26)	659 (16.64)	280 (18.74)	

LBW: low birth weight; VLBW: very low birth weight; ELBW: extremely low birth weight; NTD: new Taiwan dollars. * *p* < 0.05; ** *p* < 0.01; *** *p* < 0.001 compared with the term group.

**Table 2:** Prevalence and adjusted hazards ratio of cerebral palsy in preterm and term-born cohorts.

	Term *n* = 100,268	Preterm LBW (1500–2499 g) *n* = 19,612	Preterm VLBW (1000–1499 g) *n* = 3961	Preterm ELBW (<1000 g) *n* = 1494	*p*
CP prevalence, *n* (cases per 1000 neonatal survivors)	254 (2.53)	543 (27.69)	385 (97.20)	220 (147.26)	<0.0001
AHR ^a^ (95% CI)	1.00	11.08 (9.54–12.86)	40.40 (34.48–47.34)	62.73 (52.37–75.14)	<0.0001

CP: cerebral palsy; LBW: low birth weight; VLBW: very low birth weight; ELBW: extremely low birth weight; AHR: adjusted hazards ratio, CI: confidential interval; ^a^ = adjusted for sex, age, region, urbanization, and insured salary grade.

**Table 3:** Age at first diagnosis of CP in preterm and term-born cohorts whose CP was caused by brain insults before 4 months of age.

Cumulative Percentage	Age at First Diagnosis of CP (Year)				*p*
	Term *n* = 203	Preterm LBW (1500–2499 g) *n* = 468	Preterm VLBW (1000–1499 g) *n* = 332	Preterm ELBW (<1000 g) *n* = 186	
25%	0.67	0.75	0.75	0.75	--
50% (median)	1.33	1.08	1.12	1.08	--
75%	3.58	2.00	1.84	1.83	--
90%	6.33	3.75	3.42	3.66	--
Mean (SD)	2.41 (2.26)	1.64 (1.58)	1.58 (1.42)	1.59 (1.46)	0.0064

CP: cerebral palsy; LBW: low birth weight; VLBW: very low birth weight; ELBW: extremely low birth weight.

## Data Availability

The datasets used in the current study are available with the application and permission of NHIRD.
